# Lignin/Epoxidized Natural Rubber Compounds Based on Wet Mixing: Impact of Epoxidation Degree on the Interface of Compounds

**DOI:** 10.3390/ma18163736

**Published:** 2025-08-09

**Authors:** Hongbing Zheng, Dongmei Yue

**Affiliations:** Key Laboratory of Beijing City on Preparation and Processing of Novel Polymer Materials, Beijing 100029, China; zhenghongbing@petrochina.com.cn

**Keywords:** lignin, epoxidized natural rubber, biomaterials, compounds

## Abstract

Natural rubber (NR) possesses excellent comprehensive properties and plays an irreplaceable role in both national defense and people’s livelihood. In recent years, lignin, as a new development trend, has emerged as a reinforcing filler in natural rubber, partially replacing traditional carbon black, or serving as an antioxidant in rubber. However, lignin, a polar biomass filler, exhibits poor compatibility with non-polar natural rubber. To address the compatibility issue between the two, this paper adopts an in situ method, utilizing formic acid and hydrogen peroxide to modify natural rubber into two types of epoxidized natural rubber (ENR) with different degrees of epoxidation (E-25% and E-45%). Subsequently, through wet mixing, it is combined with a lignin aqueous solution (20 parts), and ethanol is used as a flocculant to prepare lignin/ENR composite rubber materials. Comprehensive characterization of the composite rubber materials reveals that after epoxidation modification, the interfacial compatibility between lignin and natural rubber has been significantly improved. Wet mixing also effectively enhances the dispersibility of lignin in the rubber matrix. Compared to natural rubber, the composite material with an epoxidation degree of 25% exhibits significantly superior mechanical properties and thermal stability. The tensile properties of the composite rubber increase from 29.4 MPa to 36.2 MPa, indicating the significant reinforcing effect of lignin. This study aims to investigate the effects of the epoxidation degree (25% and 45%) of epoxidized natural rubber (ENR) and the mixing method on the compatibility and reinforcement performance of composite rubber, providing a new method for preparing high-performance lignin/ENR composites.

## 1. Introduction

Natural rubber (NR) is a natural polymer compound [1], primarily composed of cis-1,4-polyisoprene [2]. NR exhibits excellent mechanical properties and high wear resistance. Its main chain contains unsaturated double bonds that are easily modifiable [3], yet it has poor resistance to thermal oxidative aging [4]. Additionally, NR possesses unique strain-induced crystallization (SIC) characteristics [5], indicating that when subjected to external forces leading to deformation, the molecular chains can align to form crystals, thereby resisting external force damage and enhancing mechanical properties. Currently, carbon black is the most widely used reinforcing filler in natural rubber applications [6]. Carbon black exhibits excellent affinity with most rubber matrices and provides superior reinforcement to rubber [7]. However, the preparation of carbon black relies on the thermal cracking of petrochemical resources [8,9,10], and its excessive use poses sustainability challenges. Therefore, developing new fillers that are renewable, environmentally friendly, and have high reinforcing capabilities has become an important direction for the sustainable development of the rubber industry [11,12,13].

Lignin is one of the most important components of plants [14], possessing environmentally friendly characteristics such as recyclability, renewability, and biodegradability [15,16]. The paper industry generates a large amount of lignin by-products annually, but they are often burned as waste [17,18], resulting in significant waste. In recent years, the research on high-value lignin has received increasing attention from both industry and academia [19]. Adding lignin to natural rubber can not only reduce costs but also enhance the mechanical properties and anti-aging properties of natural rubber [20,21]. The development and utilization of lignin/natural rubber composites not only achieve high-value utilization of lignin resources but also save raw rubber and carbon black fillers [22,23]. However, lignin contains a large number of polar groups, such as phenolic hydroxyl/alcoholic hydroxyl groups [24], which make lignin difficult to disperse well in non-polar rubber matrices [25], affecting the interfacial adhesion between the two materials and greatly hindering the application of lignin in natural rubber [26,27].

Addressing the issue of poor interfacial compatibility between lignin and natural rubber (NR), numerous researchers have attempted various strategies to resolve this problem, but most of them are based on the dry blending method [26], with relatively few reports on the wet blending method of lignin/NR. Wet blending is the process of mixing fillers with natural rubber lotion in a liquid medium. Compared to the traditional dry blending method, wet blending enables the fillers to disperse more uniformly, reduces agglomeration, and forms a more stable interfacial bond.

**Figure 1:**
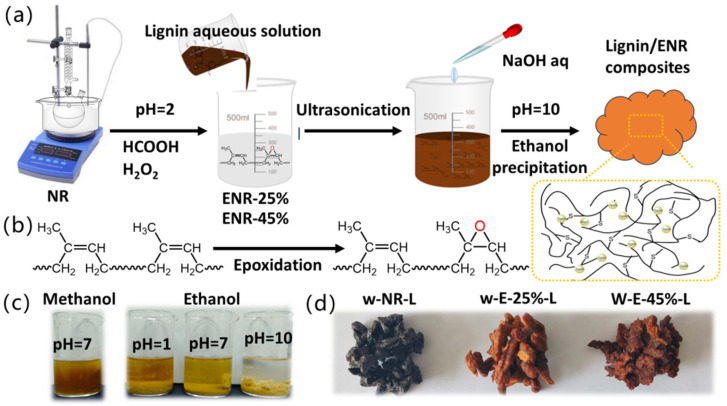
(**a**) Schematic diagram of wet mixing preparation of ENR/lignin compounds. (**b**) Epoxidation of natural rubber. (**c**) Precipitation of ENR/lignin compounds under different conditions. (**d**) Photos of ENR/lignin compounds with varying degrees of epoxy content.

This article first enhances the polarity and reactivity of natural rubber through epoxidation (ENR), while retaining its strain-induced crystallization effect [28], making it more compatible with lignin (Figure 1a,b). Subsequently, the epoxidized natural rubber and lignin are mixed via wet blending to ensure sufficient dispersion of lignin in the rubber matrix. Finally, the mixture of epoxidized natural rubber and lignin is precipitated under alkaline conditions to obtain lignin/ENR composites. The influence of different degrees of epoxidation on the properties of lignin/ENR composites was investigated, and wet and dry blending strategies were compared at the same degree of epoxidation. The results showed that lignin/ENR composites exhibited the best mechanical properties when the epoxidation degree was 25%; composites prepared using wet blending were significantly better than those prepared using dry blending. This study aims to investigate the effects of the epoxidation degree (25% and 45%) of epoxidized natural rubber (ENR) and the mixing method on the compatibility and reinforcement performance of composite rubber, providing a new method for preparing high-performance lignin/ENR composites.

## 2. Materials and Methods

### 2.1. Materials

Natural rubber latex was purchased from Xishuangbanna Tianye Rubber Company (Xishuangbanna, China) (with a solid content of 62.0%); sodium lignosulfonate was purchased from Yunnan Yunjing Forest Paper (Pu’er, China) (Sulfonic acid group content ≈ 1.55 mmol/g, Figure S1). Formic acid (HCOOH, 98.0%), hydrogen peroxide (H_2_O_2_, 30% aqueous solution), sodium hydroxide (NaOH, 99.0%), emulsifier (Dodecylphenol ethoxylates, 99%), zinc oxide (ZnO, 99.9%), stearic acid (SA, 99.0%), N-tert-butyl-2-benzothiazole sulfonamide (CBS, 99.0%), 2,2-dibenzothiazole disulfide (DM, 98.0%), and sulfur (S, 99.5%) are all analytically pure and were purchased from Shanghai Titan Science Co. Ltd. (Shanghai, China).

### 2.2. Performance Testing and Characterization

The epoxide fraction was quantified using ^1^H NMR spectroscopy (Avance 400 MHz spectrometer, Bruker, Portsmouth, Carteret, NJ, USA) at room temperature. Epoxidized natural rubber was dissolved in deuterated chloroform. The epoxide fraction was calculated by the ratio of integrated peak areas in the NMR spectrum. Peaks with chemical shifts of 2.7 and 5.2 ppm represent the epoxy protons and olefin protons in epoxidized natural rubber, respectively. The epoxide fraction was calculated using the following equation:

(1) $$Epoxide\;fraction=\frac{A_{2.7}}{A_{5.2}+A_{2.7}}\times 100\%$$

where A_2.7_ and A_5.2_ are integral peak areas at 2.7 and 5.2 ppm chemical shifts in the NMR spectra [29].

Fourier transform infrared spectroscopy (FT-IR) measurements were conducted using a Thermal Scientific Nicolet iS5 spectrometer (Perkin Elmer Inc., Waltham, MA, USA). The resolution was 4 cm^−1^, and each recorded spectrum was obtained through 32 scans, covering a wavenumber range from 4000 to 500 cm^−1^.

The morphology of lignin and lignin/ENR rubber composites was observed using scanning electron microscopy (SEM) on a Nova Nano SEM 430 instrument (FEI, Eindhoven, The Netherlands) at an acceleration voltage of 10 kV. The fracture surfaces of rubber samples for SEM were obtained by splitting the bulk samples and quenching them in liquid nitrogen. Before the observation, all samples were plated with a thin layer of gold.

After vulcanizing the rubber compounds in a standard mold, tensile tests were conducted on the vulcanized rubber in accordance with the GB/T 528-2009 standard. Prior to testing, the thickness of each specimen was measured and recorded. The tests were performed at a crosshead speed of 500 mm/min. For each sample, five parallel tests were carried out, and the average of the measurement results was selected for analysis.

Dynamic mechanical analysis (DMA) spectra of the rubber composites were obtained by using a DMA 242D dynamic mechanical analyzer (NETZSCH Gerätebau GmbH, Selb, Germany). The specimens were measured in tensile mode at a constant frequency of 1 Hz and a temperature range from −100 to 100 °C at a heating rate of 3 °C/min.

Thermo-gravimetric analysis (TGA) was performed by an STA 6000 (Perkin Elmer Inc., Norwalk, CT, USA) in the temperature range from 40 to 800 °C with a heating rate of 10 °C/min under a nitrogen atmosphere.

### 2.3. Preparation of ENR/Lignin Compounds

The epoxidation modification of natural rubber is carried out through an in situ method. First, 300 g of natural rubber latex (with a solid content of 60%) is added to a three-neck flask, diluted with water to achieve a solid content of 30%, and placed in an oil bath. Then, 20 mL of dodecylphenol ethoxylates (10% aqueous solution) is added and stirred for emulsification for 2 h, while maintaining the reaction temperature at 40 °C. During the epoxidation modification process, formic acid (30% concentration) and hydrogen peroxide (30% concentration) are added in a molar ratio of 1:0.55:1 (NR: formic acid: hydrogen peroxide). ENR emulsions with epoxidation degrees of 25% and 45% are prepared by reacting for 6 and 12 h, respectively. The pH value of the emulsion is adjusted to 7 for later use. Next, 40.0 (for ENR-45%) or 38.0 g (for ENR-25%) of sodium lignosulfonate is directly dissolved in 300 mL of water and completely dissolved through ultrasonic treatment for 30 min (20% of the rubber mass, 20 phr). The lignin solution is mixed with 100 phr of epoxidized natural rubber emulsion (E-25% and E-45%), respectively, and stirred at room temperature for 30 min. After adjusting the pH value of the lignin/ENR mixed emulsion to 10, it is slowly poured into 1000 mL of ethanol and allowed to flocculate at room temperature. After standing for 12 h, the precipitate is dried at 80 °C to obtain lignin/ENR composite materials. The dried lignin/ENR composite materials are mixed in a double-roll mill according to the following formula: 2.0 phr stearic acid, 5.0 phr zinc oxide, 1.5 phr CBS, 0.5 phr DM, and 1.5 phr sulfur (the feeding order is consistent with the formula order), while controlling the roll temperature at 50–60 °C. The mixing time is controlled within the range of 20 min. The optimal vulcanization time (T90) of lignin/ENR composite rubber is measured using a rotorless rheometer at 143 °C. Finally, vulcanization is performed using a flat vulcanizer at 143 °C, 10 MPa, and the corresponding vulcanization time. The obtained composite materials were named w-E-25%-L and w-E-45%-L, respectively. Lignin/NR composite rubber was prepared using the same method and named w-NR-L. Traditional dry mixing was performed using a double-roll mixer according to the following recipe: 100.0 phr of NR or ENR (E-25%, E-45%), 2.0 phr of stearic acid, 5.0 phr of zinc oxide, 20 phr of sodium lignosulfonate (milled at 500 rpm for 2 h), 1.5 phr of CBS, 0.5 phr of DM, and 1.5 phr of sulfur (the feeding order was consistent with the recipe or-der). Vulcanization was performed using the same method, and the obtained compo-site materials were named d-NR-L, d-E-25%-L, and d-E-45%-L, respectively.

## 3. Results and Discussion

### 3.1. Characterization of Epoxidized Natural Rubber

**Figure 2:**
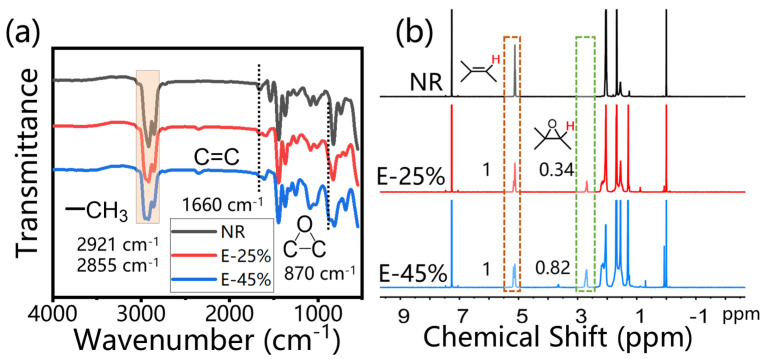
(**a**) FT-IR and (**b**) ^1^H NMR spectra of ENR with different epoxy degrees.

After epoxidation modification, the infrared spectrum and nuclear magnetic resonance spectrum of natural rubber have undergone significant changes. In FT-IR, as the degree of epoxidation of ENR increases, the number of double bonds decreases, which is reflected in the corresponding decrease in absorption intensity at 835 cm^−1^ and 1660 cm^−1^ in the infrared spectrum (Figure 2a). A new strong absorption peak appears at 870 cm^−1^, which is the asymmetric stretching vibration peak of the epoxy group [30]. As the degree of epoxidation increases, the absorption intensity of this peak also increases. In NMR, 2.7 ppm is the signal peak of protons on the carbon of the epoxy bond, and 5.2 ppm is the signal peak of protons on the carbon of the double bond [29] (Figure 2b). The degree of epoxidation can be calculated based on the peak area ratio of the two absorption peaks mentioned above. Natural rubber (NR) does not show a hydrogen spectral peak at 2.70 ppm, indicating that NR does not contain epoxy groups. As the reaction time increases, the absorption peak at 2.7 ppm gradually increases, indicating a gradual increase in the degree of epoxidation. By controlling the reaction time to 6 h and 12 h, ENR with epoxidation degrees of 25% and 45%, respectively, can be obtained. The small peak at 3.47 ppm is a characteristic proton peak on the furan group generated by the ring-opening side reaction of the epoxy group. By optimizing the reaction conditions, this side reaction can be controlled to be less than 1%.

### 3.2. Preparation of ENR/Lignin Compounds via Wet Mixing

Due to the uniform mixing of lignin and epoxidized natural rubber in the liquid state, this good dispersion is maintained during the co-precipitation process and subsequent rubber mixing and processing. Throughout the entire process, the final sedimentation is crucial. We have tried various organic solvents for sedimentation and found that alcohol solvents exhibit good sedimentation effects, with ethanol showing superior sedimentation compared to methanol, which may be related to the solubility of lignin in methanol. We also investigated the effect of pH on sedimentation. It was found that under alkaline conditions with a pH greater than 10, good sedimentation effects were exhibited (Figure 1c). Interestingly, the samples obtained after drying exhibited distinctly different colors depending on the degree of epoxidation (Figure 1d). The composite material without epoxidation and lignin appeared dark brown, close to the color of solid lignin, indicating that lignin may still exist in the natural rubber in an aggregated form. The composite material modified by epoxidation was closer to the color of lignin in aqueous solution, indicating that lignin exhibits good dispersion in epoxidized natural rubber.

### 3.3. Curing Characteristic Curve of ENR/Lignin Compounds

**Figure 3:**
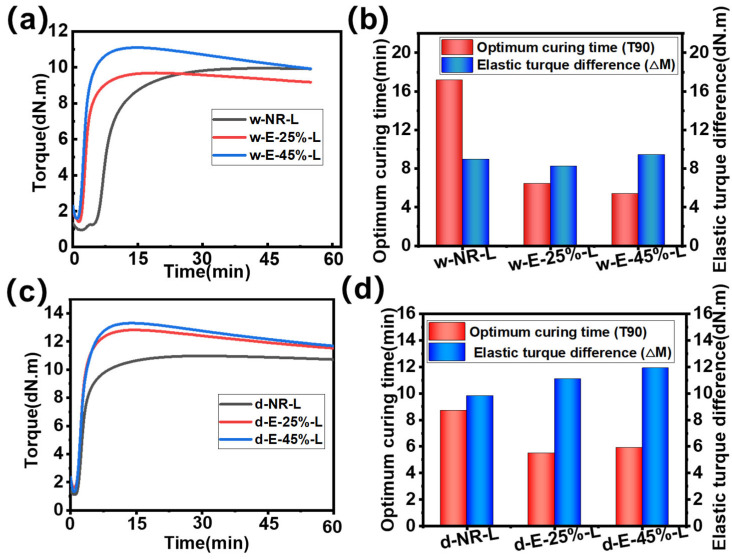
(**a**) Vulcanization curves, (**b**) optimal vulcanization time (T90), and △M (MH-ML) for wet mixing; (**c**) vulcanization curves, (**d**) optimal vulcanization time (T90), and △M (MH-ML) for dry mixing.

The curing characteristic curve of ENR/lignin compounds is shown in Figure 3, and the relevant data are summarized in Table 1. For composite materials prepared via wet mixing, as the degree of epoxidation increases, both the curing time T90 and scorch time T10 of ENR/lignin composites decrease. This is mainly due to the fact that epoxy groups promote the reaction between double bonds and sulfur [31], and epoxy bonds may also react with hydroxyl groups on lignin [32], further shortening the reaction time (Figure 3a,b). This also leads to a shortened scorch period and reduced operational safety [33]. In dry-mixed compounded rubber, T10 increases with the increase in epoxidation degree, while T90 shows a decreasing trend, which should be attributed to the differences in lignin particle size and compatibility with the rubber matrix. The minimum torque (ML), maximum torque (MH), and δ torque of lignin-filled rubber composites significantly increase with increasing epoxidation degree. ML is mainly related to the physical crosslinking between lignin and the rubber matrix before vulcanization [34], indicating stronger physical crosslinking between lignin and the rubber matrix. MH is related to the hardness and chemical crosslinking of vulcanized composite rubber [35]. When the epoxidation degree is 45%, MH increases significantly, which may be due to the higher crosslinking density of lignin-filled NR composites and the formation of a filler-rubber network between lignin and the rubber matrix. This will enhance the restriction on the mobility of rubber chains, significantly improving the strength of rubber composites. For composite materials prepared using the dry mixing method, the vulcanization time will decrease as the epoxy degree increases. However, among composites with the same epoxy degree, those prepared using the dry mixing method exhibit a higher MH value (Figure 3c,d), which may be related to the large particle size of lignin [36]. The large particle size of lignin imparts high rigidity and hardness to the composite rubber.

### 3.4. SEM Analysis of ENR/Lignin Compounds

**Figure 4:**
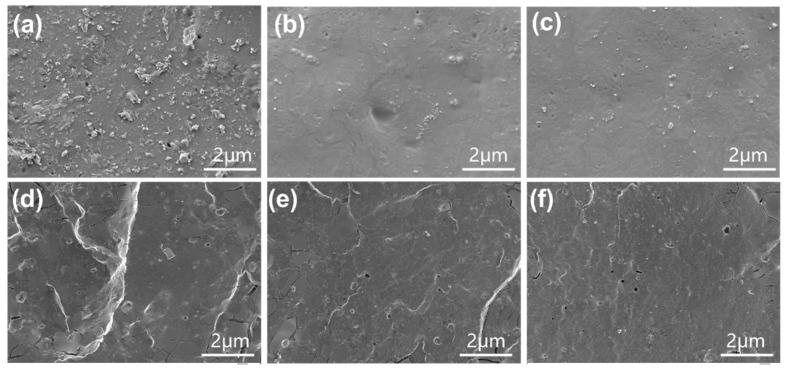
SEM images of the fractured surfaces of (**a**) w-NR-L, (**b**) w-E-25%-L, (**c**) w-E-45%-L, (**d**) d-NR-L, (**e**) d-E-25%-L, and (**f**) d-E-45%-L compounds at a magnification of 1000×.

Figure 4 presents scanning electron microscopy (SEM) images of the composite material, which are used to characterize the dispersion state of lignin in the composite material. For composite materials prepared using the wet mixing method, when natural rubber was used as the matrix, a large number of lignin particles and their outlines were clearly visible (Figure 4a–c and Figure S2). These lignin particles have irregular shapes and diameters ranging from hundreds of nanometers to several micrometers, indicating poor interfacial compatibility between lignin and NR matrix, which can affect the crosslinking density and strength of vulcanized rubber [37]. After epoxidation modification, lignin is dispersed into smaller particles. In the E-25% rubber composite material, some micron-sized lignin particles still remain in the rubber; however, these particles are significantly smaller compared to those in natural rubber. In E-45% rubber composite materials, lignin is fully dispersed at the submicron scale and exhibits high compatibility with the ENR matrix. There is a strong interfacial interaction between lignin and ENR, enabling the complete transfer of external stress from the ENR matrix to the lignin particles. Furthermore, the smaller the lignin particles, the greater the stress they can withstand, which is the fundamental reason for lignin’s reinforcing effect on rubber materials [38]. In the composite materials prepared using the dry mixing method, the particle size of lignin is relatively large, and there are many cracks in the rubber (Figure 4d–f). Although the cracks decrease after epoxy modification, they still exist, indicating that lignin particles not only have no reinforcing effect but also damage the macrostructure of the rubber matrix itself.

### 3.5. Mechanical Property Characterization of ENR/Lignin Compounds

**Figure 5:**
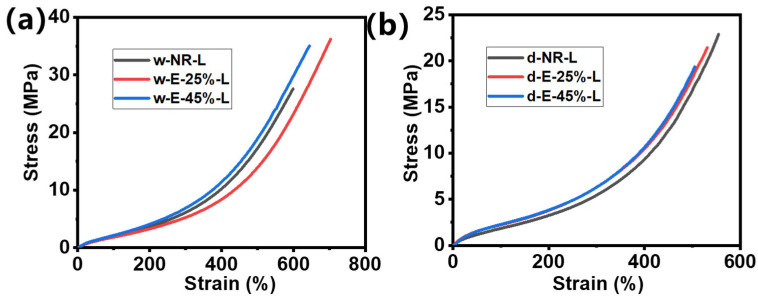
Stress–strain curves of ENR/lignin compounds using the (**a**) wet and (**b**) dry mixing methods.

The tensile stress–strain curves of lignin combined with NR and E-25%/E-45% composite rubbers are shown in Figure 5 and Figure S3. The mechanical strength of rubber composites modified with epoxy is higher than that of unmodified rubber composites. In E-25% composite rubber, the tensile strength increases from 27.5 MPa to 36.2 MPa, and the elongation at break increases from 598.8% to 703.3%; the reinforcement effect is notably enhanced (Table 2). The mechanical properties of rubber composites depend on the dispersion state of the matrix rubber and lignin, as well as the interaction between lignin and rubber. In unmodified natural rubber composites, there are numerous lignin aggregates in the non-polar rubber matrix, leading to poor interfacial interaction and an insignificant enhancement effect. After epoxy modification, lignin forms a more uniform mixture with ENR latex, greatly improving its compatibility, and lignin is dispersed at the submicron level in ENR. Under the shear force of a double-roll mixer, rubber and lignin are more tightly locked together, enhancing their interaction and achieving a high enhancement effect. At high epoxidation levels, despite the further increase in polarity of rubber and the better compatibility between lignin and the rubber matrix, the mechanical properties of high epoxidation composite materials decrease. This may be related to the influence of high epoxidation on strain-induced crystallization (SIC). The excellent mechanical properties of NR primarily stem from its SIC. Studies have shown that the self-crosslinking property of natural rubber is retained after epoxidation modification; a lower degree of epoxidation (≤20%) promotes self-crosslinking, while a higher degree of epoxidation hinders it, ultimately leading to a decrease in the mechanical properties of ENR. Compared to w-E-25%-L, w-E-45%-L exhibits better compatibility between ENR and lignin, as evident in Figure 4. However, at higher degrees of epoxidation, the influence of self-crosslinking on the mechanical properties of composite rubber becomes dominant, resulting in a reduction in the tensile strength of w-E-45%-L to 35 MPa. For composites prepared using dry mixing, their mechanical strength is significantly lower than that of composites prepared via wet mixing, with a maximum value of only 22.9 MPa, as shown by d-NR-L. After epoxidation modification, the tensile strength further decreases. The primary reason for this result is that under dry mixing conditions, lignin cannot be fully dispersed; large particles of lignin not only cannot withstand stress but also damage the macrostructure of rubber, leading to a reduction in the tensile strength of composite rubber. Simultaneously, the self-crosslinking property decreases after epoxidation modification, and as the degree of epoxidation increases, the tensile strength further decreases.

### 3.6. Dynamic Thermomechanical Characterization of ENR/Lignin Compounds

**Figure 6:**
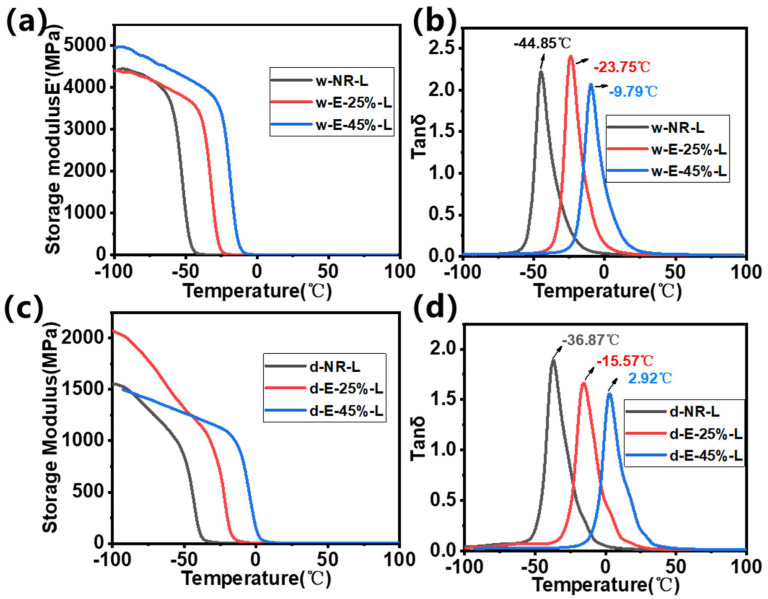
(**a**,**c**) Storage modulus (*E’*) and (**b**,**d**) Tan *δ* of ENR/lignin compounds using the (**a**,**b**) wet and (**c**,**d**) dry mixing methods.

The dynamic mechanical behavior of ENR/lignin composites was investigated using dynamic thermodynamic analysis. Figure 6 illustrates the relationship between the storage modulus (*E’*) and loss factor (tan *δ*) of lignin/ENR composites after heat treatment, as well as their dependence on temperature. The storage modulus can be regarded as the elastic modulus of rubber composites, while the loss factor is related to the dissipation of energy in the form of heat [39]. As shown in Figure 6a, the initial storage modulus (*E’*_0_) of the epoxy-modified composites increases, especially at higher epoxy degrees. *E’*_0_ represents the stiffness of the composite [40], and its increase is attributed to the enhanced lignin–rubber interaction. Within the temperature range from −100 to −60 °C, the storage modulus exhibits a linear relationship with temperature. This behavior originates from natural rubber remaining in the glassy state within this temperature range, and the filler does not enhance its properties. As the temperature increases, the w-NR-L curve initially drops sharply, while w-E-25%-L and w-E-45%-L decrease at higher temperatures. This is related to the glass transition temperature (*T*_g_) of the three composite rubbers.

In the Tan *δ* curve, the abscissa of the peak represents the *T*_g_ of the composite material [41] (Figure 6b). The *T*_g_ of NR typically ranges from −70 to −60 °C. After epoxidation modification, due to the influence of the epoxy bond structure, the glass transition temperature increases, and the *T*_g_ of E-45% is generally between −30 and −20 °C [15]. Upon the addition of lignin, the glass transition temperature of all composite rubbers shows an upward trend, attributed to the rigid structure of lignin. The *T*_g_ of sodium lignosulfonate used in this study is 138.4 °C. The *T*_g_ of w-NR-L is −44.9 °C, which increases to −23.8 °C at a lower epoxy degree and further increases to −9.8 °C at a higher epoxy degree. In ENR/lignin composites, an increase in the *T*_g_ indicates the formation of a more tightly cross-linked structure within the composite material. After epoxy modification, the compatibility of lignin with the rubber matrix is improved, enabling lignin to disperse better in the rubber and form covalent bonds with the epoxy rubber, thereby enhancing the cross-linking network and hindering the migration of rubber molecular chains. In the Tan *δ* curve, w-E-25%-L exhibits a higher loss peak, which is related to the small amount of lignin aggregation observed in SEM images. Under the action of tensile force, slippage occurs within the lignin aggregates, leading to an increase in modulus loss. As the epoxy degree increases, this phenomenon disappears, indicating that epoxy modification can improve the compatibility of lignin with rubber and enhance the rubber properties under wet mixing conditions. The initial storage modulus of the composite prepared using the dry mixing method is significantly lower than that of the composite prepared using the wet mixing method, indicating poor interfacial compatibility between lignin and ENR in the dry mixing strategy (Figure 6c). Additionally, the *T*_g_ of the composite prepared using the dry mixing method is significantly higher than that of the composite prepared using the wet mixing method (Figure 6d), which may be related to the rigidity of the composite caused by larger lignin particles.

### 3.7. Characterization of Thermal Stability of ENR/Lignin Compounds

**Figure 7:**
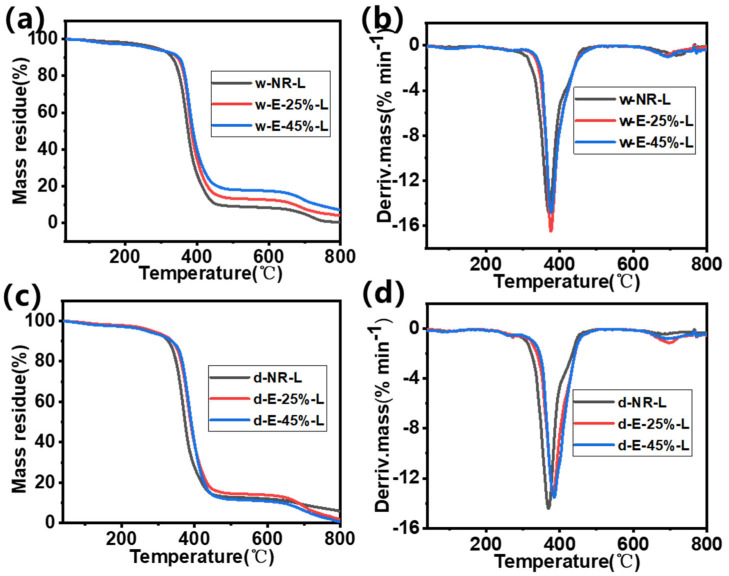
(**a**,**c**) TGA and (**b**,**d**) DTG thermograms of ENR/lignin compounds using the (**a**,**b**) wet and (**c**,**d**) dry mixing methods.

The thermal stability of ENR/lignin composites was evaluated through thermogravimetric analysis (TG) and differential thermal analysis (DTG) curves (Figure 7 and Figure S4). As the degree of epoxidation increases, the resistance to degradation of lignin/rubber composites enhances, as shown in Table 3 [42]. When the epoxy degree is 25%, its decomposition temperature can be increased from 375.2 to 385.0 °C; when the epoxy degree is 45%, its decomposition temperature further increases to 389.3 °C, indicating that the thermal stability of vulcanized rubber after epoxy modification is significantly improved. Lignin exhibits excellent thermal stability [21], attributed to its antioxidant properties, where its phenolic hydroxyl groups prevent oxidation [24]. Through epoxy modification and wet mixing strategies, the compatibility of lignin with natural rubber is greatly enhanced, enabling lignin to be more effectively embedded in rubber as smaller particles, significantly improving the thermal stability of the composite rubber. On the differential thermal analysis (DTG) curve, the highest weight loss peak of w-E-45%-L occurs at 376.2 °C, which may be related to the aggregation of lignin particles observed in SEM images. However, the thermal stability of composite materials prepared under different mixing strategies did not exhibit significant differences.

## 4. Conclusions

In summary, two types of epoxidized natural rubber (ENR) with different degrees of epoxidation (25% and 45%) were prepared using the in situ method, and ENR/lignin composite rubber materials were successfully prepared through the wet mixing method. The results showed that the higher the degree of epoxidation, the better the interfacial compatibility between ENR and lignin. However, the highest mechanical properties were achieved when the degree of epoxidation was 25%, as an excessively high degree of epoxidation may affect the “strain-induced crystallization effect” of natural rubber. Additionally, we demonstrated that the composite materials prepared using the wet mixing method are significantly superior to those prepared using the dry mixing method. The wet-mixing strategy utilizing 25% epoxidized NR establishes a novel paradigm for high-performance biocomposites, demonstrating 23.2% enhancement in tensile strength relative to conventional methods.

## Figures and Tables

**Table 1:** The curing characteristic parameters of ENR/lignin compounds.

Samples	T10 (min)	T90 (min)	ML (dN·m)	MH (dN·m)	△M (MH-ML) (dN·m)
w-NR-L	5.0	17.2	1.0	10.0	9.0
w-E-25%-L	2.3	6.4	1.4	9.7	8.3
w-E-45%-L	1.9	5.4	1.6	11.1	9.5
d-NR-L	1.6	8.7	1.2	10.9	9.3
d-E-25%-L	1.7	5.7	1.7	12.8	11.1
d-E-45%-L	2.0	6.0	1.4	13.3	11.9

**Table 2:** Mechanical properties parameters of ENR/lignin compounds.

Samples	Tensile Strength (MPa)	Elongation at Break (%)	Stress at 100% (MPa)	Stress at 300% (MPa)
w-NR-L	27.5 ± 0.6	598.8 ± 19.3	1.0 ± 0.02	2.2 ± 0.03
w-E-25%-L	36.2 ± 0.5	703.3 ± 25.4	1.0 ± 0.03	2.1 ± 0.06
w-E-45%-L	35.0 ± 0.7	644.5 ± 17.6	1.1 ± 0.04	2.5 ± 0.03
d-NR-L	22.9 ± 0.6	554.7 ± 19.1	0.9 ± 0.05	1.9 ± 0.03
d-E-25%-L	21.1 ± 1.3	531.2 ± 32.5	1.3 ± 0.04	2.3 ± 0.02
d-E-45%-L	19.2 ± 1.6	505.2 ± 38.6	1.2 ± 0.03	2.3 ± 0.05

**Table 3:** Thermal degradation of ENR/lignin compounds.

Samples	Decomposition Temperature (°C)
w-NR-L	375.2
w-E-25%-L	385.0
w-E-45%-L	389.3
d-NR-L	373.9
d-E-25%-L	389.1
d-E-45%-L	390.9

## Data Availability

The raw data supporting the conclusions of this article will be made available by the authors on request.

## Supplementary Materials

The following supporting information can be downloaded at: https://www.mdpi.com/article/10.3390/ma18163736/s1, Figure S1: (a) FT-IR, (b) DSC, (c) TG, and (d) DTG curves of lignosulfonate sodium; Figure S2: SEM image of lignosulfonate sodium; Figure S3: Stress-strain curves of NR, E-25%, and E-45%; Figure S4: (a) TG and (b) DTG curves of NR, E-25%, and E-45%; Table S1: Mechanical properties parameters of NR, E-25%, and E-45%; Table S2: Thermal degradation of NR, E-25%, and E-45%.

## References

[1] Zhang G., Jiang Y., Wang S., Zhang Y. (2023). Influence of a Novel Coupling Agent on the Performance of Recovered Carbon Black Filled Natural Rubber. Compos. Part B Eng..

[2] Yamashita S., Takahashi S. (2020). Molecular Mechanisms of Natural Rubber Biosynthesis. Annu. Rev. Biochem..

[3] Martinez J.M.M. (2006). Natural Rubber by a Rubber Man. Mater. Today.

[4] Bawn C.E.H. (1964). The Chemistry and Physics of Rubber-like Substances. Polymer.

[5] Mai T., Yasui T., Tanaka R., Masunaga H., Kabe T., Tsunoda K., Sakurai S., Urayama K. (2024). Unraveling Non-Uniform Strain-Induced Crystallization Near a Crack Tip in Natural Rubber. Adv. Sci..

[6] Du X., Zhang Y., Pan X., Meng F., You J., Wang Z. (2019). Preparation and Properties of Modified Porous Starch/Carbon Black/Natural Rubber Composites. Compos. Part B Eng..

[7] Dwivedi C., Manjare S., Rajan S.K. (2020). Recycling of Waste Tire by Pyrolysis to Recover Carbon Black: Alternative & Environment-Friendly Reinforcing Filler for Natural Rubber Compounds. Compos. Part B Eng..

[8] Saengdee L., Phinyocheep P., Daniel P. (2020). Chemical Modification of Natural Rubber in Latex Stage for Improved Thermal, Oil, Ozone and Mechanical Properties. J. Polym. Res..

[9] Mei J., Liu W., Huang J., Qiu X. (2019). Lignin-Reinforced Ethylene-Propylene-Diene Copolymer Elastomer via Hydrogen Bonding Interactions. Macromol. Mater. Eng..

[10] Zhou Y., Fan M., Chen L., Zhuang J. (2015). Lignocellulosic Fibre Mediated Rubber Composites: An Overview. Compos. Part B Eng..

[11] Roy K., Debnath S.C., Pongwisuthiruchte A., Potiyaraj P. (2021). Recent Advances of Natural Fibers Based Green Rubber Composites: Properties, Current Status, and Future Perspectives. J. Appl. Polym. Sci..

[12] Kazemi H., Mighri F., Park K.W., Frikha S., Rodrigue D. (2022). Hybrid Nanocellulose/Carbon Nanotube/Natural Rubber Nanocomposites with a Continuous Three-dimensional Conductive Network. Polym. Compos..

[13] Noguchi T., Bamba Y., Miura T., Iwamoto R., Endo M., Isogai A. (2021). Cellulose-Nanofiber-Reinforced Rubber Composites with Resorcinol Resin Prepared by Elastic Kneading. Macromol. Mater. Eng..

[14] Ragauskas A.J., Beckham G.T., Biddy M.J., Chandra R., Chen F., Davis M.F., Davison B.H., Dixon R.A., Gilna P., Keller M. (2014). Lignin Valorization: Improving Lignin Processing in the Biorefinery. Science.

[15] Li M., Zhu L., Xiao H., Shen T., Tan Z., Zhuang W., Xi Y., Ji X., Zhu C., Ying H. (2022). Design of a Lignin-Based Versatile Bioreinforcement for High-Performance Natural Rubber Composites. ACS Sustain. Chem. Eng..

[16] Liu Z.H., Li B.Z., Yuan J.S., Yuan Y.J. (2022). Creative Biological Lignin Conversion Routes toward Lignin Valorization. Trends Biotechnol..

[17] Collins M.N., Nechifor M., Tanasă F., Zănoagă M., McLoughlin A., Stróżyk M.A., Culebras M., Teacă C.-A. (2019). Valorization of Lignin in Polymer and Composite Systems for Advanced Engineering Applications—A Review. Int. J. Biol. Macromol..

[18] Xia Z., Li J., Zhang J., Zhang X., Zheng X., Zhang J. (2020). Processing and Valorization of Cellulose, Lignin and Lignocellulose Using Ionic Liquids. J. Bioresour. Bioprod..

[19] Lizundia E., Sipponen M.H., Greca L.G., Balakshin M., Tardy B.L., Rojas O.J., Puglia D. (2021). Multifunctional Lignin-Based Nanocomposites and Nanohybrids. Green Chem..

[20] Ikeda Y., Phakkeeree T., Junkong P., Yokohama H., Phinyocheep P., Kitano R., Kato A. (2017). Reinforcing Biofiller “Lignin” for High Performance Green Natural Rubber Nanocomposites. RSC Adv..

[21] Barana D., Ali S.D., Salanti A., Orlandi M., Castellani L., Hanel T., Zoia L. (2016). Influence of Lignin Features on Thermal Stability and Mechanical Properties of Natural Rubber Compounds. ACS Sustain. Chem. Eng..

[22] Xue B., Wang X., Yu L., Di B., Chen Z., Zhu Y., Liu X. (2020). Self-Assembled Lignin-Silica Hybrid Material Derived from Rice Husks as the Sustainable Reinforcing Fillers for Natural Rubber. Int. J. Biol. Macromol..

[23] Chang B.P., Gupta A., Muthuraj R., Mekonnen T.H. (2021). Bioresourced Fillers for Rubber Composite Sustainability: Current Development and Future Opportunities. Green Chem..

[24] Parvathy G., Sethulekshmi A.S., Jayan J.S., Raman A., Saritha A. (2021). Lignin Based Nano-Composites: Synthesis and Applications. Process Saf. Environ. Prot..

[25] Hosseinmardi A., Amiralian N., Hayati A.N., Martin D.J., Annamalai P.K. (2021). Toughening of Natural Rubber Nanocomposites by the Incorporation of Nanoscale Lignin Combined with an Industrially Relevant Leaching Process. Ind. Crops Prod..

[26] Qiu J., Yuan S., Xiao H., Liu J., Shen T., Tan Z., Zhuang W., Ying H., Li M., Zhu C. (2023). Study on Lignin Amination for Lignin/SiO2 Nano-Hybrids towards Sustainable Natural Rubber Composites. Int. J. Biol. Macromol..

[27] Jiang C., Shen H., Bi X., Wang Z., Yao M., Wu Y., Zhang L., Yu P. (2022). A Green Dual-Phase Carbon-Silica Nanohybrid Derived from Black Liquor Lignin for Reinforcing Styrene-Butadiene Rubber. Compos. Sci. Technol..

[28] Burfield D.R., Lim K., Law K. (1984). Epoxidation of Natural Rubber Latices: Methods of Preparation and Properties of Modified Rubbers. J. Appl. Polym. Sci..

[29] Singh M., Mohd Rasdi F.R. (2019). Colloidal Properties of Epoxidized Natural Rubber Latex Prepared via Membrane Separation Technology. J. Rubber Res..

[30] Gan L.H., Ng S.C. (1986). Kinetic Studies of the Performic Acid Epoxidation of Natural Rubber Latex Stabilized by Cationic Surfactant. Eur. Polym. J..

[31] Pongdong W., Nakason C., Kummerlöwe C., Vennemann N. (2015). Influence of Filler from a Renewable Resource and Silane Coupling Agent on the Properties of Epoxidized Natural Rubber Vulcanizates. J. Chem..

[32] Jiang C., He H., Yao X., Yu P., Zhou L., Jia D. (2015). In Situ Dispersion and Compatibilization of Lignin/Epoxidized Natural Rubber Composites: Reactivity, Morphology and Property. J. Appl. Polym. Sci..

[33] Datta J., Parcheta P. (2017). A Comparative Study on Selective Properties of Kraft Lignin–Natural Rubber Composites Containing Different Plasticizers. Iran. Polym. J..

[34] Wang H., Liu W., Huang J., Yang D., Qiu X. (2018). Bioinspired Engineering towards Tailoring Advanced Lignin/Rubber Elastomers. Polymers.

[35] Mohamad Aini N., Othman N., Hussin M., Sahakaro K., Hayeemasae N. (2019). Hydroxymethylation-Modified Lignin and Its Effectiveness as a Filler in Rubber Composites. Processes.

[36] Boeriu C.G., Bravo D., Gosselink R.J.A., Van Dam J.E.G. (2004). Characterisation of Structure-Dependent Functional Properties of Lignin with Infrared Spectroscopy. Ind. Crops Prod..

[37] Mohamad Aini N.A., Othman N., Hussin M.H., Sahakaro K., Hayeemasae N. (2020). Lignin as Alternative Reinforcing Filler in the Rubber Industry: A Review. Front. Mater..

[38] Thakur V.K., Thakur M.K., Raghavan P., Kessler M.R. (2014). Progress in Green Polymer Composites from Lignin for Multifunctional Applications: A Review. ACS Sustain. Chem. Eng..

[39] Barana D., Orlandi M., Zoia L., Castellani L., Hanel T., Bolck C., Gosselink R. (2018). Lignin Based Functional Additives for Natural Rubber. ACS Sustain. Chem. Eng..

[40] Rolere S., Cartault M., Sainte-Beuve J., Bonfils F. (2017). A Rheological Method Exploiting Cole-Cole Plot Allows Gel Quantification in Natural Rubber. Polym. Test..

[41] Sarkawi S.S., Aziz A.K.C., Rahim R.A., Ghani R.A., Kamaruddin A.N. (2016). Properties of Epoxidized Natural Rubber Tread Compound: The Hybrid Reinforcing Effect of Silica and Silane System. Polym. Polym. Compos..

[42] Košíková B., Gregorová A., Osvald A., Krajčovičová J. (2007). Role of Lignin Filler in Stabilization of Natural Rubber–Based Composites. J. Appl. Polym. Sci..

