# Supplementary material for: Lignin/Epoxidized Natural Rubber Compounds Based on Wet Mixing: Impact of Epoxidation Degree on the Interface of Compounds
Source: Materials (Basel). 2025 Aug 9;18(16):3736. doi: 10.3390/ma18163736 (PMC12387426; doi:10.3390/ma18163736)
Supplement: Supplementary file 1 [file materials-18-03736-s001.zip › materials-3786692-supplementary.pdf]

Supporting Information

Lignin/epoxidized natural rubber compounds based on wet mixing: Impact of epoxidation degree on the interface of compounds

Hongbing Zheng and Dongmei Yue*

Beijing University of Chemical Technology, Beijing 100029, PR China; Key Laboratory of Beijing City on Preparation and Processing of Novel Polymer Materials, Beijing 100029, PR China; zhenghongbing@petrochina.com.cn

Correspondence: Dongmei Yue：yuedm@mail.buct.edu.cn

Supporting Figures

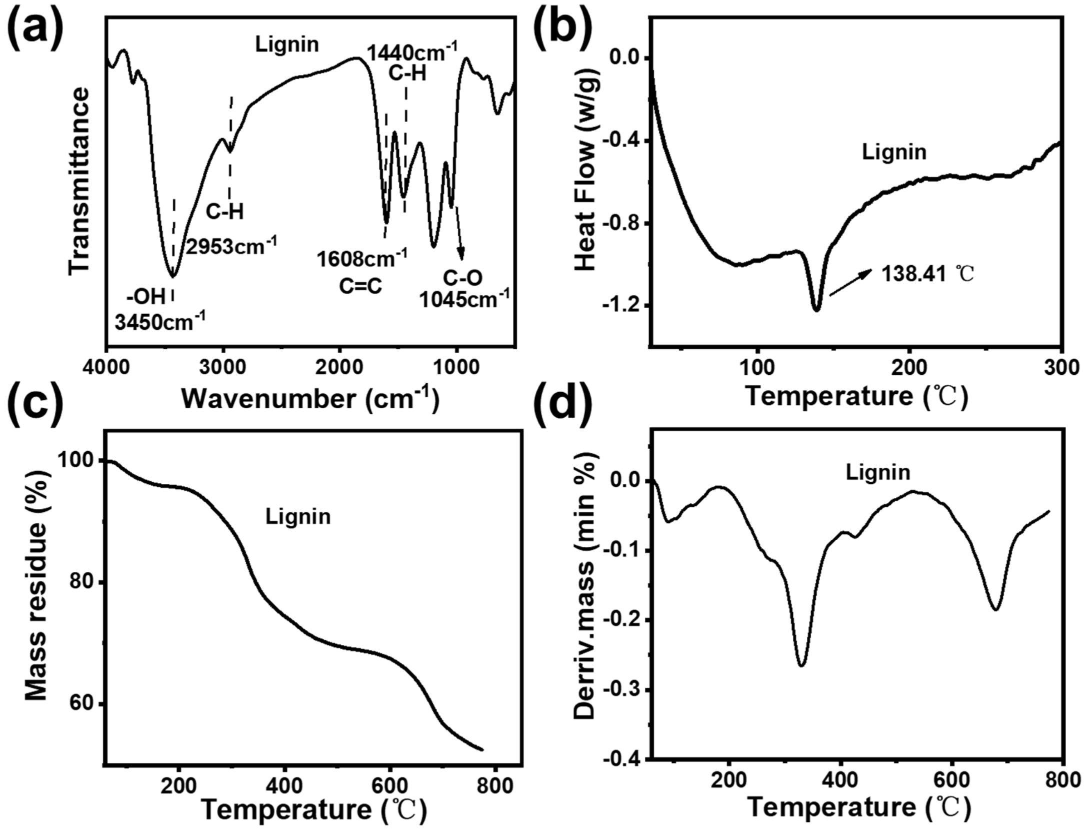

Figure. S1 (a) FT-IR, (b) DSC, (c) TG, and (d) DTG curves of lignosulfonate sodium.

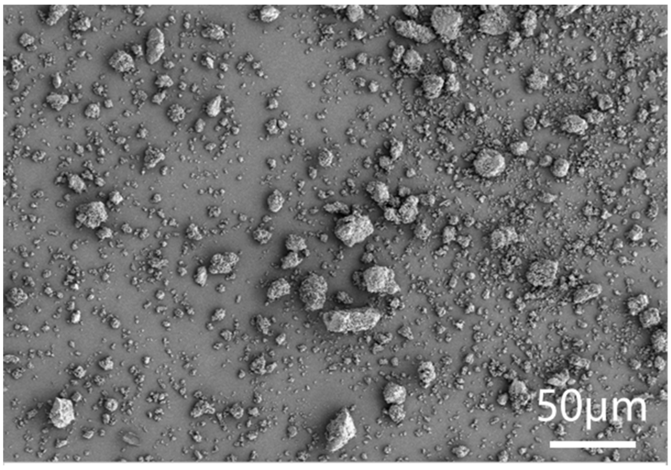

Figure. S2 SEM image of lignosulfonate sodium.

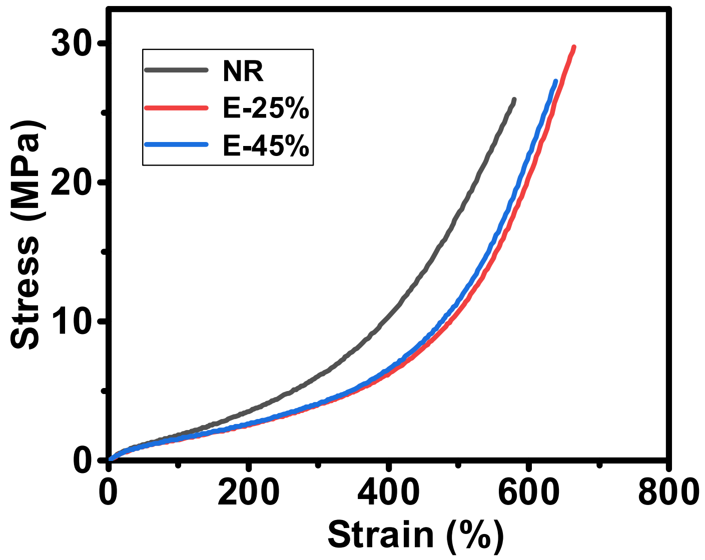

Figure. S3 Stress-strain curves of NR, E-25%, and E-45%.

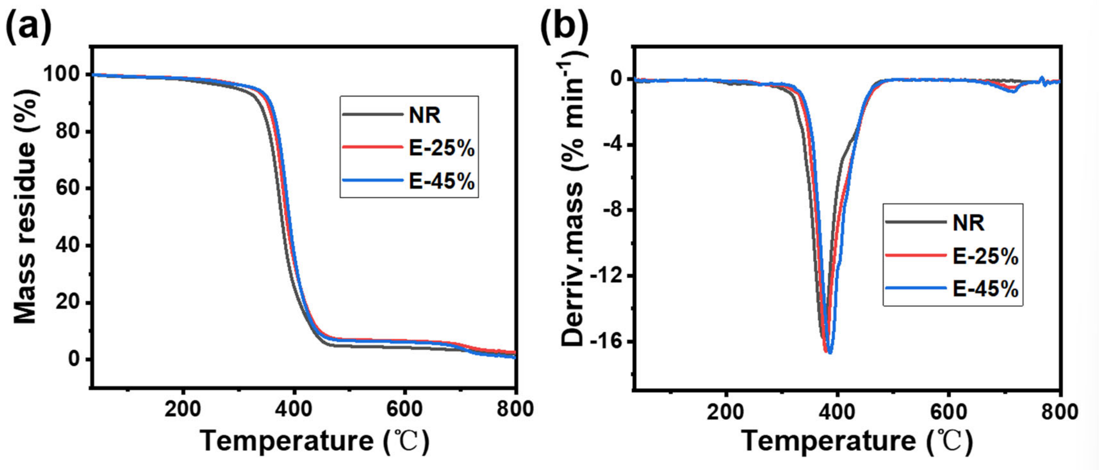

Figure. S4 (a) TG and (b) DTG curves of NR, E-25%, and E-45%.

Supporting Tables

Table S1. Mechanical properties parameters of NR, E-25%, and E-45%.

Samples	Tensile strength (MPa)	Elongation at break (%)	Stress at 100% (MPa)	Stress at 300% (MPa)
NR	25.9 ± 0.5	579.2 ± 21.3	1.0 ± 0.04	2.1 ± 0.03
E-25%	29.8± 0.4	667.5± 24.3	0.9± 0.03	1.7 ± 0.05
E-45%	27.3 ± 0.8	638.3 ± 22.7	0.9 ± 0.05	1.7 ± 0.02

Table S2. Thermal degradation of NR, E-25%, and E-45%.

Samples	Decomposition Temperature (^{o}C)
NR	377.2
E-25%	385.8
E-45%	390.7.3
